# Community pharmacists’ history taking practice in responding to acute uncomplicated cystitis: A simulated patient study from Sudan

**DOI:** 10.1371/journal.pone.0343006

**Published:** 2026-02-17

**Authors:** Riham M. Hamadouk, Esra D. Albashair, Einass M. Alshareif, Ali Awadallah Saeed, Bashir A. Yousef

**Affiliations:** 1 Department of Pharmacy Practice, Faculty of Pharmacy, University of Khartoum, Khartoum, Sudan; 2 Department of Clinical Pharmacy and Pharmacy Practice, Faculty of Pharmacy, Almughtaribeen University, Khartoum, Sudan; 3 Department of Pharmacology, Faculty of Clinical and Industrial Pharmacy, The National University-Sudan, Khartoum, Sudan; 4 The Mycetoma Research Center, University of Khartoum, Khartoum, Sudan; 5 Department of Clinical Pharmacy and Pharmacology, Ibn Sina National College of Medical Studies, Jeddah, Saudi Arabia; 6 Department of Pharmacology, Faculty of Pharmacy, University of Khartoum, Khartoum, Sudan; Curtin University Bentley Campus: Curtin University, AUSTRALIA

## Abstract

**Background:**

Today, community pharmacists’ responsibilities have expanded beyond the traditional role to include the management of minor ailments. Acute uncomplicated cystitis is one of the most prevalent medical conditions seen in primary healthcare and can be managed by community pharmacists (CPs).

**Objectives:**

To evaluate community pharmacists’ history-taking practice when responding to patients with acute uncomplicated cystitis.

**Methods:**

A cross-sectional simulated patient study was conducted from November 2022 to January 2023 in Khartoum locality targeting only pharmacists. Six trained female students played the simulation in which they pretended to have the clinical symptoms of acute uncomplicated cystitis and requested treatment for their condition. The Interactions during the simulation were documented immediately in a data collection form after each visit. Descriptive statistics were used to report the study outcomes.

**Results:**

A total of 238 community pharmacies were visited. The majority of the pharmacists were female. The mean of the number of questions that were asked by the pharmacists was 1 (SD = 1.6) questions. Among the studied pharmacists, 45.4% asked at least one question during patient assessment. The most asked question was if the patient has a fever, representing 61 (25.6%) CPs, followed by if there is vaginal discharge, representing 38 (16%) CPs. In response to scenario 47 (19.7%) CPs decided to refer the patient to a physician, and 45 (18.9%) of the CPs advised the patient to increase water intake.

**Conclusions:**

The study has revealed a poor history-taking practice towards acute uncomplicated cystitis during patient assessment. Further studies exploring pharmacists’ involvement in patient assessment are required. Strategies to improve community pharmacists’ practice, like continuing pharmacy education and providing a national guideline regarding patient assessment should be considered seriously.

## Introduction

Community pharmacies are established as the most accessible facilities in the health care system, visited by large numbers of people who seek their services. This can reflect the fundamental role that the community pharmacist can play in patients’ education and improving public health [1]. Community pharmacists are highly educated and possess valuable knowledge [2]. The core of their professional role is a combination of a dispensing process and patient counseling, which is considered a patient-centered service rather than a drug product-centered service [3]. Today, community pharmacists’ responsibilities have expanded beyond the traditional role; they involve managing chronic diseases, promoting healthy lifestyles, treating minor ailments, optimizing medication use, and acting as a source of advice for both patients and healthcare providers [4].

Minor ailments are ‘common or self-limiting or uncomplicated conditions which can be diagnosed and managed without medical intervention’ [5]. Even though minor ailments don’t need medical intervention and will resolve on their own, these conditions remain a major source of medical and financial burden in developed and developing countries [6]. Many patients who visit community pharmacies rely on community pharmacists’ judgment to treat their minor and acute ailments, and this depends on the pharmacist’s knowledge and awareness of the treatment guidelines for these illnesses [7]. They can identify minor ailments, provide suitable over-the-counter medication, and effectively advise patients regarding their conditions [8].

Safety netting is one of the actions that should be performed by community pharmacists in managing minor illnesses. Safety netting refers to the information provided by the healthcare members to the patient or his/her caregiver during the consultation regarding what the patient should do if their condition changes or the given medication/s fails to improve patient health [9]. Several studies have investigated community pharmacists’ management of minor ailments [10–12], and all have revealed inadequate information gathering. Improper information gathering by community pharmacists will result in poor patient assessment, which might lead to misdiagnosis and consequently provide inappropriate medications that can be unnecessary or even harmful to the patient [13].

Cystitis is one of the conditions that can be managed by community pharmacists. It is a group of urinary symptoms that include frequent urination, increased urgency, and dysuria (painful voiding). Sometimes the urine becomes cloudy and may have a strong smell which indicates bacterial infection, and this necessitates referral [14]. It is more common in women, and this is thought to be a result of anatomical variations, as they have a shorter urethra and lack the prostatic secretions [15]. Acute uncomplicated cystitis is one of the most prevalent medical conditions seen in primary healthcare, affecting more than 50% of all women during their lifetime, with an estimation suggesting that approximately one-third of females will have had at least one episode of cystitis by the age of 24 years [16].

In many developing countries including Sudan, the patients’ first contact with the healthcare system is through community pharmacies, as accessibility to the healthcare facilities is affected by the economic status [17]. In these countries, community pharmacists can play a significant role in reducing economic burden by guiding the patients’ decisions, especially in treating minor conditions by identifying the correct diagnosis, preventing unnecessary medications, and decreasing the medical cost [18]. In Sudan, there are no local guidelines to guide the practice of community pharmacists in managing minor ailments, and according to the Sudan Pharmaceutical Country Profile, there is no national policy to control antibiotic resistance or prohibit antibiotic dispensing without a prescription [19].

Acute uncomplicated cystitis is very common, and most of cases will resolve without treatment, despite that many patients request therapy to relieve their symptoms [20]. According to a systematic review, in developing countries urinary tract infections are among the most common infectious diseases, and it approximately affecting 32% of the sub-Saharan African populations. Also, the diagnosis of urinary tract infections is not always straightforward, which can lead to antimicrobial resistance [21]. Community pharmacists are in a prime position to manage urinary tract infections more effectively [22], especially through antimicrobial stewardship [23]. As mentioned in the literature, in many developing countries, patients can purchase antibiotics from community pharmacies without prescriptions [24–27]. Also, a study conducted in South Africa has reported inappropriate dispensing of antibiotics without prescriptions to patients with urinary tract infections [28]. And it is well known that the inappropriate use of antibiotics is the major cause of antimicrobial resistance [29]. Therefore, we decided to conduct this study to evaluate community pharmacists’ practice when responding to patients complaining of acute uncomplicated cystitis, concentrating on pharmacists’ history-taking practice and whether the pharmacist offers safety netting.

## Methods

### Study design

This was a cross-sectional study in accordance with the STROBE statement [30]. The simulated patient method was implemented in this study to observe how community pharmacists respond to patients with cystitis symptoms, as it is internationally used to assess the actual practice, and it minimizes the Hawthorne effect. The study was carried out in Khartoum locality. Table 1. summarizes the details of the study methodology.

**Table 1:** Summary of study methodology.

Methodological component	Description
**Study Design**	Cross-sectional simulated patient (SP) study conducted in accordance with the STROBE reporting guidelines.
**Study Setting**	Community pharmacies in Khartoum locality, Sudan.
**Scenario Development**	One scenario of acute uncomplicated cystitis designed by the research team to assess history-taking practices.
**Scenario Validation**	Scenario reviewed and validated by four faculty experts (two physicians, two clinical/pharmacy practice pharmacists) for clinical appropriateness and essential questioning requirements. Consensus indicated no need for referral.
**Study Population**	Registered community pharmacies in Khartoum locality (N = 587).
**Sampling Strategy**	Simple random sampling using the national pharmacy list. Sample size calculated using n = N/ (1 + N(e²)) with 5% margin of error, resulting in 238 pharmacies.
**Simulated Patients (SPs)**	Six trained female fourth-year pharmacy students.
**SP Training Procedures**	Two-week structured training covering scenario presentation, standardized responses, and accurate checklist completion. Training included supervised rehearsals, recorded sessions for feedback, and standardization under physician supervision.
**Pilot Testing**	Conducted in 30 pharmacies (not included in final sample) to evaluate SP readiness, scenario fidelity, and assessment form reliability.
**Assessment Tool**	Structured checklist with 10 history-taking items and 3 response-related items, developed using Symptoms in the Pharmacy. Content validated by three pharmacy practice experts. Reliability demonstrated with Cronbach’s alpha = 0.869.
**Data Collection Process**	Each pharmacy visited once by an SP who requested to speak to a pharmacist. Encounters conducted only when pharmacies were not crowded. No audio recordings used; immediate post-visit documentation minimized recall bias.
**Data Management**	Data entered and cleaned using SPSS software.
**Statistical Analysis**	Descriptive statistics for demographic variables and SP-reported outcomes. Chi-square test used to assess associations between categorical variables and pharmacists’ history-taking performance. Significance threshold p < 0.05.
**Ethical Considerations**	Ethical approval obtained from the Faculty of Pharmacy, University of Khartoum, Sudanese General Directorate of Pharmacy, and Ministry of Health. Written consent obtained from pharmacists two months prior to data collection to protect the covert design. Confidentiality maintained throughout.

### Scenario

One scenario was developed by the researchers, a case of acute uncomplicated cystitis. The scenario was designed to assess the questioning process employed by community pharmacists when managing acute uncomplicated cystitis, and only one scenario was applied, as we excluded the other possible scenarios for not serving the scope of our study. The scenario was constructed to demonstrate a case that does not necessitate referral. As cystitis is one of the most common infections in women [31], the simulation was performed by females. The study scenario was validated by four members of the University of Khartoum, two physicians, and two pharmacists specialized in clinical pharmacy and pharmacy practice. They were chosen according to their knowledge and experience in clinical and practice settings to determine what constitutes a reasonable outcome regarding community pharmacists’ practice in this scenario, and what the essential questions they should ask. The members specified that the scenario did not warrant the recommendation of medical referral. Also, an agreement was made by the four members on the questions suggested by the researchers to be asked by the community pharmacists. They also deemed it vital for community pharmacists to recommend nonpharmacologic advice, such as increasing water intake. In the scenario, the simulated patient (SP) will enter the pharmacy and request a pharmacist, then she will complain of discomfort when passing urine with frequency and urgency to urinate. No further information will be provided to the pharmacist unless he/she asks, and the questions asked by the pharmacist were answered agreeing with the patient characteristics mentioned in the scenario. The details of the scenario are in Table 2.

**Table 2:** Details of the simulation scenario.

Scenario
The simulated patient enters the pharmacy and asks for a pharmacist, then she will complain of discomfort in urination, with frequency and urgency.
If the pharmacist asks the simulated patient, the following information will be provided:
- She is 25 years old. - She is not married or pregnant. - The symptoms started 1 day ago. - No fever, nausea/vomiting, loin pain, or tenderness. - No blood in the urine or vaginal discharge. - She does not have any chronic diseases or urologic abnormalities (diabetes, stones). - No medication was used to treat the condition.

### Sample size and sampling

The community pharmacies included in this study were selected using simple random sampling from a list obtained from the Sudanese General Directorate of Pharmacy. This list contains all registered community pharmacies in the Khartoum locality. The sample size was calculated using the equation n = N/1 + N(e)^2^, where N is the study population (587 pharmacies), and (e) is an error margin of 0.05. The equation is based on a degree of variability (p) of 0.5 and a 95% confidence interval. Using the equation, the sample size for this study (n) was 238 pharmacies, which were selected using the Excel random number generator. These pharmacies were visited only once to present the study scenario.

### Assessment form

To assess community pharmacists’ history-taking practice regarding acute cystitis, the authors designed a data collection form to document the information obtained from simulated visits. It was developed in accordance with the information provided in Symptoms in the Pharmacy: A Guide to the Management of Common Illnesses [13]. It consists of two parts, the first includes ten assessment questions that should be asked by the community pharmacist when taking a patient’s history to reach to appropriate decision about the condition, either to treat or refer. The second part contains three questions regarding the community pharmacist’s responses to the scenario. Details in Table 3.

**Table 3:** Assessment form.

Assessment items	Coding	
	Not Discussed	Discussed
1. How long have the symptoms been present? 2. Is the patient pregnant? 3. Are there other medical conditions? (diabetes, stones) 4. Does the patient have vomiting or nausea? 5. Does the patient have a fever? 6. Does the patient suffer from loin pain or tenderness? 7. Is blood present in the urine? 8. Is vaginal discharge accompanied by the symptoms? 9. Has this condition occurred before? 10. Did the patient use any medications to treat this condition?	0 0 0 0 0 0 0 0 0 0	1 1 1 1 1 1 1 1 1 1
**The response of the CP to the simulated scenario**		
Does the pharmacist recommend increasing water intake?		
Does the pharmacist refer the patient to a physician before assessing the patient?		
Does the pharmacist provide safety netting?		

Three academic members in the Faculty of Pharmacy, University of Khartoum, who had excellent experiences in pharmacy practice approved the assessment form. Moreover, the assessment form has been piloted in thirty community pharmacies to test its validity. The reliability analysis of the assessment questions showed a Cronbach’s alpha coefficient of 0.869.

### Data collection

To collect the data, six fourth-year female students from the Faculty of Pharmacy volunteered to be the simulated patients (SPs). Before the data collection, the researchers explained the principles of the methodology to the SPs and trained them for two weeks on aspects related to the presentation of the scenario, tailoring their answers to specific pharmacist questions, and carefully filling the checklist that was used as an assessment form. The training sessions were recorded to enable the SPs to notice the defects in their performance and modify them according to the researchers’ adjustments. Several rehearsals were also made to ensure consistency in the illness simulation, which were supervised by a physician. A pilot study was carried out to make the SPs more familiar with the data collection process and confirm their readiness to deliver the scenario. The SPs were accompanied by one of the researchers to ascertain that every participant undergoes the same simulation with the same steps and circumstances to provide a consistent basis for assessment. A total of thirty community pharmacies were visited during the pilot study (five visits for each SP), and these pharmacies were not included in the study sample. The researcher also audio-recorded all the pilot visits. The audios were reviewed by the remaining researchers to further standardize the SPs’ performance and make sure the scenario is portrayed with fidelity.

The study was conducted from November 2022 to January 2023. As the study targeted only pharmacists, in each visit the SP requested the pharmacist first before performing the simulation. On entering the pharmacy, and once the pharmacist was identified, the SP presented the rehearsed scenario and provided information upon the community pharmacist’s request based on the scripted scenario. Also, weekly rehearsals were conducted during the period of data collection to guarantee the uniformity and consistency of SPs’ performance. Further, to avoid the impact of crowdedness in pharmacists’ practice, the SPs approached the pharmacies only when they were empty. The SPs contact only one pharmacist at each community pharmacy, even if there is more than one pharmacist working at the pharmacy.

Immediately following each encounter, and to minimize the recall bias, the SPs recorded the response of the community pharmacists regarding the scenario in an assessment form that was designed to evaluate their practice. The assessment form was designed to be filled out with yes or no to minimize distortions in results due to faulty memories, as an audio recording was not used due to ethical issues.

### Data management and analysis

The Statistical Package for Social Sciences software, version 26.0 (IBM SPSS Inc., Chicago, IL) was used for data analysis. Descriptive statistics were used to describe the demographic characteristics of the pharmacists under study and the information obtained from the data collection forms. The results were illustrated as tables that contain frequencies and percentages. The ten assessment questions could be either “yes” which was given a score of 1, or “no” which was given a score of 0. The maximum total score that can be attained for the assessment practice will be ten. Scores between 0 and 3 were considered poor, scores between 4 and 6 were considered fair, and scores between 7 and 10 were considered good. The chi-square test, multivariable logistic regression model and were utilized to test the association and correlation between categorical variables such as gender, age, years of experience as a community pharmacist, and the highest qualification, and the pharmacist’s practice in history taking. The results of regression analysis are expressed as adjusted odds ratios (aORs) with 95% confidence intervals (CIs), with p-values of less than 0.05 were considered statistically significant.

### Ethical approval

This research was approved by the Ethical Committee of the Faculty of Pharmacy, University of Khartoum, Khartoum, Sudan (FPEC-32–2022). Additional approval was obtained from the Sudanese General Directorate of Pharmacy and the scientific research authority, Ministry of Health. Moreover, as SP studies involve deception, which can be taken as violating the expectations of honesty, and to protect the scientific validity of the study, the researchers collected written informed consent from all participating community pharmacists. The informed consent was collected two months before the study, as the time span between prior information and execution of the visits is crucial. If this time span is too short, the covert study design is jeopardized. The consent informed the pharmacists that they will be part of an observational study and that their practice will be assessed. Also, it clarifies that the study will not cause any damage to the participants. No information regarding the name of the study or the method of the assessment was provided. Confidentiality was maintained throughout the study.

## Results

### Demographic characteristics of community pharmacists

As planned, the SPs visited 238 pharmacies in Khartoum locality. Almost 65% of the community pharmacists were females, and 43.3% of the participants were aged between 26–30 years old. The majority of the community pharmacists in this study (84.9%) were bachelor’s degree holders, while 57.6% of them had experience in community pharmacy settings ranging between 2–5 years. More details are in Table 4.

**Table 4:** Demographic characteristics of the community pharmacists (n = 238).

Variable	Category	Frequency (%)
Age (Years)	21-25 26-30 31-35 36-40	87 (36.6) 103 (43.3) 35 (14.7) 13 (5.5)
Gender	Male Female	84 (35.3) 154 (64.7)
The highest qualification of the community pharmacist	B. Pharm Master	202 (84.9) 36 (15.1)
Years of experience as a community pharmacist	<2 2-5 6-10 >10	50 (21) 137 (57.6) 42 (16.6) 9 (3.8)

### The community pharmacists’ practice regarding the simulated scenario

Regarding community pharmacists’ history-taking practice, 45.4% of the CPs asked at least one question during patient assessment. The mean number of questions asked was 1.0 ± 1.6, with a median of 0 questions and a range of 0–7 questions, none of the CPs asked all ten questions that related to the assessment of cystitis. The most asked question was if the patient has a fever, which was asked by 61 (25.6%) CPs, followed by if there is a vaginal discharge, which was asked by 38 (16%) CPs, and the duration of the symptoms, which was asked by 37 (15.5%) CPs. While none of the community pharmacists asked if the patient had vomiting or nausea. In response to the scenario, 47 (19.7%) community pharmacists decided to refer the patient to a physician, and 45 (18.9%) of the community pharmacists advised the patient to increase water intake. Further information is in Table 5.

**Table 5:** Actions and advice of community pharmacists (CPs) in response to the scenario and the medications dispensed during the simulated patient study.

Community pharmacist’s history-taking practice	Number of CPs (%)	
	Yes	No
**Patient assessment items**		
How long have the symptoms been present? Is the patient pregnant? Are there other medical conditions? (diabetes, stones) Does the patient have vomiting or nausea? Does the patient have a fever? Does the patient suffer from loin pain or tenderness? Is blood present in the urine? Is vaginal discharge accompanied by the symptoms? Has this condition occurred before? Did the patient use any medications to treat this condition?	37 (15.5) 19 (8) 4 (1.7) 0 (0) 61 (25.6) 27 (11.3) 1 (0.4) 38 (16) 24 (10.1) 32 (13.4)	201 (84.5) 219 (92) 234 (98.3) 238 (100) 177 (74.4) 211 (88.7) 237 (99.6) 200 (84) 214 (89.9) 206 (86.6)
Mean number of questions asked	1.0 ± 1.6	
Median (range)	0 (0–7)	
**Actions done by CPs in response to the simulated scenario**		
Referring to a doctor directly without patient assessment Recommendation of increasing water intake Provision of safety netting (n = 191)	47 (19.7) 45 (18.9) 0 (0)	191 (80.3) 193 (81.1) 191 (80.3)
**Name of dispensed medication**	**Frequency (%)**	
No medication Potassium Citrate Coli urinal® (Piperazine/Hexamine/Khellin) Urinex® (Alpha pinene/Camphene/Borneol) A combination of medications that included an antibiotic	30 (12.6) 36 (15.13) 5 (2.1) 2 (0.84) 165 (69.3)	

In this study, 30 (12.6%) CPs decided not to dispense any medication in response to the scenario, while at least one antibiotic alone or in combination with other medications was dispensed in 69.3% of the encounters. When an antibiotic was not dispensed, potassium citrate was the most dispensed medication representing 15.1% of the dispensed medications. See Table 5.

The practice of community pharmacists in assessing patients with acute uncomplicated cystitis was poor. No significant association was found between the community pharmacists’ practice and their demographic characteristics (Table 6). Furthermore, the multivariable logistic regression analysis demonstrated that none of the community pharmacists’ demographic characteristics were significantly associated with their history-taking practice (Table 7).

**Table 6:** Relationship between the practice of community pharmacists’ history taking practice and their demographics.

Variables		Community pharmacists’ practice			P-value
		Poor	Fair	Good	
Gender	Male Female	74 145	8 8	2 1	.220
Age categories (Years)	21-25 26-30 31-35 36-40	79 95 33 12	5 8 2 1	3 0 0 0	.469
Years of experience as a community pharmacist	<2 2-5 6-10 >10	47 125 39 v8	2 10 3 1	1 2 0 0	.934
Highest qualification	B. Pharm Master	184 35	15 1	3 0	.440

P-value < 0.05 is considered statistically significant.

**Table 7:** Multivariable logistic regression analysis of demographic factors associated with the practice of community pharmacists’ history taking practice.

Variable	aOR (95% CI)	P value
**Gender**		
Male	Reference	**--------**
Female	0.721 (0.51 - 10.15)	0.809
**Age (years)**		
21-25	Reference	--------
26-30	0.197 (0.247–2.564)	0.742
31-35	0.235 (0.57–4.974)	0.634
36-40	0.345 (0.19–4.697)	0.533
**The highest qualification of the community pharmacist**		
B. Pharm	Reference	--------
Master	0.322 (0.038–2.746)	0.305
**Years of experience as a community pharmacist**		
<2	Reference	--------
2-5	2.137 (0.525–8.707)	0.289
6-10	2.708 (0.311–23.618)	0.367
>10	3.807 (0.113–12.760)	0.456

**Note:** aOR = adjusted odds ratio; CI = confidence interval. P-value < 0.05 is considered statistically significant.

## Discussion

Globally, there is an increasing demand for the provision of healthcare services in community pharmacies [32]. Many developed countries have effectively incorporated community pharmacy professionals into a range of public health initiatives including minor ailments management [33]. In Sudan, most patients depend on community pharmacists to treat minor ailments, as the deteriorated economic status hinders patients’ ability to access other healthcare facilities.^7^ Reflecting on that and as a part of the expanding role of pharmacists in comprehensive patient care, the pharmacist should conduct an appropriate interview with each patient regarding his/her medical history for symptoms of the present complaint as an attempt to arrive at the right diagnosis [34].

The current study evaluated community pharmacists’ history-taking practice regarding acute uncomplicated cystitis in Khartoum, Sudan. The results revealed poor history-taking practice. When comparing the findings, our result was consistent with what was found by S. Zawahir et al. in their study that assessed the community pharmacy staff’s response to symptoms of common infections, whereas in the uncomplicated urinary tract infection scenario, the patient’s history taking was also poor. Additionally, in the same study, the other scenarios which include sore throat, common cold, and diarrhea, insufficient information gathering was also observed during history taking, and the overall history-taking practice for the study was poor [35].

Similarly, in accordance with our findings, a simulated patient study conducted in Lebanon aimed to evaluate antibiotic prescribing by community pharmacists using a scenario of acute uncomplicated cystitis, the results revealed unsatisfactory history-taking practice, as the mean number of questions asked by community pharmacists was 3.1 (SD = 2.27) questions [36]. Many simulated studies were conducted with scenarios different from our scenario had also mentioned the inadequate patient history taking practice. A study from Ethiopia that explored the management of minor ailments by community pharmacy staff has found that only 26.2% had requested additional details regarding the patient’s symptoms and medical and medication history [37]. Also, a study by W. Saengcharoen et al. that aimed to assess the community pharmacist and client factors that affect the practice in managing upper respiratory tract infection, reported that among the studied pharmacists, the history taking was poor [38].

Further, the results of the Malaysian study which used a case of cough in their scenario, revealed that none of the participating community pharmacists had inquired sufficient patient information that helps in identifying the possible cause of cough before recommending a therapy plan [39]. Moreover, the findings from the study that evaluated the management of acute gastroenteritis by community pharmacists conducted in Qatar showed that none of the pharmacists during the SP encounters had assessed the patient’s medical history [40].

In contrast, in a study that assessed diarrhea treatment and counseling by community pharmacists in Iraq, the results revealed that the history-taking practice in the study was satisfactory [41]. While Sejal Parekh et al. have reported that most patients who complained of urinary tract infection symptoms were successfully assessed and managed by community pharmacists upon using a specific guiding leaflet for community pharmacy settings [16]. Evidence from several countries increasingly supports the important role of community pharmacists in assessing and managing acute uncomplicated urinary tract infections [22,42]. In fact, this practice has been implemented in many regions across the globe, including New Zealand [43], UK [44,45], Australia [46,47], and in some states across the USA [48,49], and it was supported either by clinical management protocols or appropriate tools and education. With respect to that, it is worth mentioning that there is no readily available national minor ailment scheme in Sudan. Additionally, no well-defined national standards are available for community pharmacists to follow regarding patients’ self-care [50], which might contribute to the current pharmacists’ practice in this study.

History taking is an important constituent of pharmaceutical care [38]. In fact, in self-medication in community pharmacies, for the pharmacist to provide medicines initially, he/she should assess the patient, and the first step of the assessment is information gathering [51]. The studied community pharmacists were mainly interested in asking about fever, which is an indicator of other complications in the upper urinary tract [31]. Only a few pharmacists asked about pregnancy and the presence of other medical conditions.

Asking about pregnancy status is crucial in the management of cystitis, as urinary tract infections have a high prevalence in pregnancy and are known to increase the risk of pregnancy complications affecting both maternal and neonatal health [52]. In addition, this study revealed high dispensing of antibiotics, including fluoroquinolones [53]. This class of antibiotic is pregnancy category C, and it cannot be dispensed to a pregnant patient unless the prescriber weighs the risks versus the benefits, as it might affect the fetus’s health [54]. Other comparable studies had also shown very low inquiry of pregnancy status during history taking [35,36,55].

Questioning regarding other medical conditions the patient has is very important. In our case, it is known that urinary tract infections are common among many chronic conditions and may be associated with the presence of an underlying disease [56], such as diabetes, where the compromised immune system and the high glucose level increase the risk of infections [57]. Unfortunately, only four pharmacists asked if the patient had other medical conditions.

Our findings indicate that almost 70% of the community pharmacists dispensed antibiotics in response to the scenario. This high rate of antibiotic dispensing without prescription was noticed in several simulated studies conducted in different parts of the world [36,55,58]. This is perhaps due to community pharmacies are typically business oriented. A. Salim et al. have already mentioned that Sudanese community pharmacists sell antibiotics for commercial interest, along with the absence of legislations that control the dispensing practices at community pharmacies [59].

Other medications that were dispensed during the simulated encounters were not mentioned in any of the guidelines. The alkalinizing agent potassium citrate, which was dispensed in this scenario, can provide symptomatic relief, although there is not enough data to support its use in cases of acute uncomplicated cystitis [14].

In considering that acute uncomplicated cystitis has an infectious origin and might require a physician’s assessment to prescribe the suitable antibiotic regimen [60], it is worth mentioning that few community pharmacists (19.7%) decided to directly refer the patient to a physician. This very low rate of referral was also seen in a study from Lebanon [36].

Nonpharmacological advice, like drinking large quantities of fluids should be suggested to patients with cystitis, as theoretically may help promote bladder voiding, which is thought to help in flushing the infecting bacteria out of the bladder [14]. In the present study, the recommendation of increasing water intake was made by only 18.9% of the community pharmacists, and safety netting was not provided by any community pharmacist. In Sudan, S. Mohamed et al. have reported that 93.7% of the community pharmacists said they ask the patient about his/her symptoms when responding to the patient’s symptoms [50]. Thus, an appropriate inquiry about patient history was expected as we targeted only pharmacists, however, our results revealed poor practice.

We believe that neither our simulated patients’ performance nor the lack of counseling time affected the practice of the community pharmacists, as they were well-trained and approached the pharmacists after ensuring that the pharmacies were empty. Therefore, the possible driver of this poor practice might be due to the lack of clinical knowledge and training, especially the process of managing minor ailments, which includes history taking unguided by a specific protocol. Another possible driver includes heavy workload in community pharmacies, insufficient motivation to provide minor ailment services, and patients’ perception that pharmacists have only a dispensary role in a community setting [61]. Enhancing pharmacists’ knowledge and competencies is fundamental in improving patient outcomes, and the key component for this is through continuing pharmacy education programs [62,63]. A study from Sudan conducted in 2022 showed that community pharmacists are interested in educational programs, especially those focusing on common diseases, and declared that these programs would improve their knowledge [63], which reflects the need to implement such programs to improve pharmacists’ practice [64].

This study has some limitations. The generalizability of the findings of the study to the whole country cannot be made, as it is conducted only in Khartoum locality—an urban setting—and may not reflect pharmacist practices in rural or remote regions. Also, the results cannot be generalized to other diseases as it discussed only a case of acute uncomplicated cystitis, and pharmacists’ responses to other cases may vary. Further, the absence of audio recordings required reliance on simulated patient recall, which introduces the potential for recall bias and may affect the completeness of the captured interactions. Also, the study did not include any qualitative component, which limits the ability to explore pharmacists’ reasoning processes, communication strategies, and clinical decision-making in depth. Lastly, certain pharmacists’ factors that may affect their performance, like training courses and previous experience in clinical settings, were not covered in the study.

## Conclusion

The presence of a pharmacist in the community pharmacy will provide an appropriate patient assessment and accurate therapy for minor ailments. This study has revealed a poor history-taking practice towards acute uncomplicated cystitis during patient assessment, and safety netting was not provided by any pharmacist. The study highlights a significant gap in pharmacists’ skills and knowledge regarding patient assessment. There is a need to ensure that community pharmacists assess the patients properly. Thus, further studies exploring pharmacists’ involvement in patient assessment on a national scale should be encouraged. Also, to ensure that students learn more about patient assessment, it is essential to incorporate patient assessment training in undergraduate pharmacy curricula. Continuing pharmacy education programs are fundamental for improving community pharmacists’ skills and knowledge, especially programs with more focus on patient assessment and minor ailments management. Furthermore, stakeholders should monitor community pharmacists’ practice and implement minor ailments management protocols to guide community pharmacists’ practice.
